# Future of cancer immunotherapy using plant virus-based nanoparticles

**DOI:** 10.2144/fsoa-2019-0001

**Published:** 2019-07-25

**Authors:** Erum Shoeb, Kathleen Hefferon

**Affiliations:** 1 Cell & Systems Biology, University of Toronto, 25 Willcocks St, Toronto, ON, M5S 1A1, Canada

**Keywords:** cancer therapy, computational modeling, drug delivery, epitope, monoclonal antibodies, nanotechnology, plant virus, virus-like particles

## Abstract

Immunotherapy potentiates a patient’s immune response against some forms of cancer, including malignant tumors. In this Special Report, we have summarized the use of nanoparticles that have been designed for use in cancer immunotherapy with particular emphasis on plant viruses. Plant virus-based nanoparticles are an ideal choice for therapeutic applications, as these nanoparticles are not only capable of targeting the desired cells but also of being safely delivered to the body without posing any threat of infection. Plant viruses can be taken up by tumor cells and can be functionalized as drug delivery vehicles. This Special Report describes how the future of cancer immunotherapy could be a success through the merger of computer-based technology using plant-virus nanoparticles.

## Lay abstract

The nonpathogenic nature of plant viral nanoparticles makes them an ideal choice for therapeutic applications such as cancer. Understanding the molecular mechanisms behind the immune response to cancer has facilitated the use of nanotechnology as an effective cancer therapy. Biologically active self-replicating plant virus particles can be introduced to the bloodstream of the human body and used as effective drug delivery vehicles. This Special Report describes how a combination of computer-based technology and plant-virus nanoparticles can assist in cancer immunotherapy.

Nanotechnology is a recently emerging science with promising impacts for the medical field. Cancer therapies including surgery, radiation and chemotherapy have largely improved over the years; however, cancer remains a challenge for patients, their families, healthcare providers and researchers. Cancer is a disease of uncontrolled cell growth and results in the spread of abnormal cells [1]. Although the human immune system is capable of fighting against diseases, cancer cells divide and accumulate mutations rapidly, thus decreasing the natural immune system’s ability to target cancerous cells [2]. Previously, inadequate knowledge of the immune system has slowed our ability to stop cancer progression. Understanding the basic mechanisms behind the human immune response against cancer cells, in addition to utilizing the benefits of nanotechnology, is an approach that could advance effective cancer therapy.

The cancer immunosurveillance process produces cells of the immune system to identify and attach to cancer-specific target cell antigens. As an antibody attaches to the antigen, it activates the immune system machinery to initiate target cell destruction. DNA sequences of a number of antigens expressed by cancer cells and recognized by host T cells are now known [3].

Naturally occurring viruses can invade a host organism’s immune system to ensure their own survival and reproduction. Viruses have evolved to attack specific hosts and cell types, and some are capable of integrating their genome into their plant or animal hosts [4,5]. Virus-like particles (VLPs) can be utilized as nanoparticles, as safe and economical tools for therapeutic purposes such as combating cancer [6]. These structures are devoid of viral genetic material, hence are not capable of replicating or causing any infection. Empty VLPs are thus potentially capable of carrying the genetic information required for therapy [7]. The idea of using assemblies of plant-virus capsid protein as VLPs has revolutionized the value of virus-based therapy, as VLPs based on human viruses can be considered to be a safety risk (Figure 1) [8].

**Figure 1.**
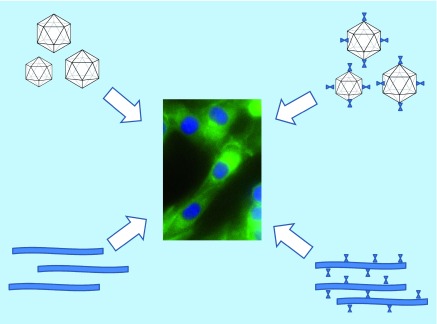
Plant virus nanoparticles can be functionalized for cancer immunotherapy. Virus-like particles based on plant viruses (left) and highly functionalized nanoparticles (right) can be used to stimulate an immune response or deliver drug payloads to cancer cells. Top of figure displays icosahedral empty VLPs (top left) and functionalized VLPs (top right). Bottom depicts naked rod-shaped plant virus nanoparticles (bottom left) and functionalized rod-shaped nanoparticles (bottom right). Photo depicts cancer cells that are to be targeted by plant virus nanoparticles. Green illustrates localization of immunofluorescent antibodies directed toward cellular vimentin, blue illustrates 4’, 6-diamidine-2'-phenylindole dihydrochloride (DAPI)-stained nuclei. VLP: Virus-like particles.

In cancer treatments, plant VLPs can be introduced to the body via the bloodstream and reach target cells through the circulation [9,10]. The targeting of cancer cells is often based on information regarding tumor cell surface receptors that are known to be overexpressed due to abnormal cell–cell signaling [11,12]. The size and shape of plant virus capsid proteins can be as small as 10 nm in diameter, an ideal size for circulation in the bloodstream and highly capable for the transportation and release of a ‘payload’ to target cells for oncolytic therapy [13].

## Use of plant virus-based nanoparticles

A number of virus vectors have been tested for cancer treatment with positive preliminary results, such as the animal virus adenovirus, and the plant virus Cowpea mosaic virus (CPMV) [14,15]. Plant viruses have already been utilized extensively for vaccine development, and thus provide a valuable alternative for drug delivery [16,17]. The successful employment of plant viruses in cancer treatment has been demonstrated using Hibiscus chlorotic ringspot virus [18], Red clover necrotic mosaic virus [19] and Tomato bushy stunt virus [20–22]. Plant virus capsid proteins offer the advantage of uniformity with respect to size and shape, and the ability to self assemble into highly repeating nanostructures. Plant VLPs can also exhibit structurally defined chemical attachment sites, tolerance against high temperature and pH, nucleases and proteases found in the intracellular environment, as well as a cargo capacity that is suitable for transporting drugs to the affected area. The fact that plant viruses are noninfectious to humans makes them a valuable but inexpensive alternative therapeutic nanoparticle for cancer therapy applications. These biodegradable and nontoxic capsid proteins can carry a desired payload and control nanoparticle presentation to target cells with more precision than any synthetic or artificial nanoparticle [23]. Nanoparticles based on plant viruses also can possess immunostimulatory properties to fight cancer cells. For example, Cowpea mosaic virus nanoparticles were also used for therapeutic activity in mice [24,25]. Similarly, Papaya mosaic (PapMV) nanoparticles have been shown to be effective for cancer immunotherapy [26,27].

## Formulation of plant virus nanoparticles

In general, plant nanoparticles may be coated with complement, neutralizing antibodies and other immune proteins that can associate and form a corona around the nanoparticle, directing it for clearance before it even reaches the target tissue. As a result, a plant virus nanoparticle such as Tobacco mosaic virus (TMV) has a short half-life of only 3½ min in mice who have not been exposed previously to the virus. To slow down rapid degradation and clearance in the human body, plant virus nanoparticles can be coated with a ‘self’ protein such as serum albumin (SA) to camouflage them from immune recognition and increase their circulation time. Gulati *et al.* [28] used animal studies to find that TMV nanoparticles coated with a high coverage of SA and short PEG linkers were optimal for preventing antibody recognition. Even TMV-specific antibodies were not able to recognize SA-coated TMV nanoparticles. When these nanoparticles were taken up into macrophages, SA was recycled whereas TMV was sent to the cell’s lysosome by intracellular trafficking, thus explaining why only antibodies toward TMV could be detected. The work is also important as many smokers harbor antibodies to TMV. Plant viral nanoparticles are also under investigation for their properties in the bloodstream. Pitek *et al*. [29] have used a mouse model to develop several methodologies to study the impact of VNP–protein corona complexes on both target cell recognition and clearance by phagocytes. The authors plan to use the insight gleaned from these studies to construct new plant virus VLP formulations.

Other plant viral nanoparticles that are icosahedral in shape, such as CPMV, have also been shown to have antitumor properties when weekly injected directly into the intraperitoneal (ip.) space in mice with disseminated tumors. A slow release formulation of CPMV nanoparticles would be highly desirable over weekly administrations. As CPMV has an overall negative charge, it was tested for its’ ability to co-assemble with positively charged dendrimers. Czapar *et al.*, [30] found that CPMV-G4 (CPMV and polyamidoamine generation 4 dendrimer) molecules are capable of forming aggregates that are based on both salt concentration as well as electrostatic interactions. These properties facilitate CPMV formulations to be tailored for different durations of nanoparticle release. The authors were able to demonstrate that a single release of CPMV-G4 was as effective at reducing tumor growth as weekly administrations of soluble CPMV; this is likely due to the increased stability of CPMV in the ip. space leading to reversed immunosuppression and prolonged immune stimulation in ovarian cancer mouse models. CPMV-G4 thus offers advantages for treating patients in actual clinical settings.

## Entry & internalization into cells

Although the surface of plant virus nanoparticles can be functionalized so that they can be preferentially taken up by cancer cells, native plant viruses themselves have been shown to enter mammalian cells in the absence of any targeting moieties [31]. Recently [32], Sesbania mosaic virus (SeMV) nanoparticles (NPs) bioconjugated with fluorophores was used to examine their ability to enter various types of mammalian cells. The authors found that SeMV was capable of entering HeLa, HepG2, MDA-MB-231 and NIH/3T3 cells; it has preference for MDA-MB-231 cells. Like TMV and CPMV, SeMV NPs interact predominantly with vimentin, exposed on the surface of mammalian cells. Vimentin is a cytoskeletal protein that is responsible for cellular architecture and has been demonstrated to act as a host attachment protein for a variety of pathogens (Dave and Bayless, 2014). Tumor cells exhibit increased amounts of surface vimentin. Icosahedral viruses such as CPMV and SeMV become internalized into mammalian cells through the endocytosis pathway, and blocking studies using antibodies specific to vimentin illustrated that this surface protein is important for plant virus internalization (Vardhan *et al*., 2018).

## Drug delivery mechanism

Plant viral nanoparticles are recognized as a highly suitable system for delivering drugs to tissues affected by cancer (Table 1). The fact that the permeability of plant virus nanoparticles can be altered by changing the pH and ionic strength is a tremendous advantage for their use as a drug delivery platform. Alemzadeh *et al.*, [33] have successfully demonstrated loading and delivery of the drug doxorubicin (Dox) using VLPs of Johnson grass chlorotic stripe mosaic virus in mice [29]. Similarly, TMV has been employed for drug-delivery and imaging of Dox in an animal cancer model. Results were very promising in terms of slow tumor growth and improved chances of survival. Drug delivery to breast cancer cells has also been reported using TMV nanoparticles [34]. TMV–cisplatin conjugates (TMV–cisPt) have facilitated the treatment of ovarian cancer [35]. The use of TMV-based nanoparticles was demonstrated for the treatment of non-Hodgkin’s lymphoma [36]. Investigations based on Potato virus X (PVX) were conducted by Lee *et al*. [37] to develop a dual-functional system of the anticancer drug, Dox, incorporated within PVX nanoparticles. This dual system was successful in delaying tumor growth in B16F10 melanoma and improved chances of survival when used as a vaccine. Furthermore, PVX was demonstrated as a carrier for drug loading and targeted delivering of Dox for ovarian, breast and cervical cancer in mice [38]. TMV based delivery of the drug phenanthriplatin has also been demonstrated by Czapar and Steinmetz [30]. TMVs carrying approximately 2000 phenanthriplatin moieties were superior in performance than free phenanthriplatin in a mouse model of breast cancer.

**Table 1.** Summary of plant virus delivery systems described in this report.

Plant virus	Capsid structure	Delivery system	Biologic delivered	Study (year)
Cowpea mosaic virus	Icosahedral	Empty virus-like particles	Not applicable	Czapar *et al.* (2018)
Hibiscus chlorotic ringspot virus	Icosahedral	Virus coat protein cage assembled around anticancer drug cargo	Doxorubicin	Ren *et al.* (2007)
Red clover necrotic mosaic virus	Icosahedral	Virus coat protein cage assembled around anticancer drug cargo	Doxorubicin	Ren *et al.* (2010)
Tomato bushy stunt virus	Icosahedral	Virus coat protein cage encapsulates anticancer drug or drug is decorated with drug on surface of cage	Not available	Matsuura (2018)
Papaya mosaic virus	Rod shaped	Assembled virus-like particle	No drug necessary	Lebel *et al.* (2016)
Tobacco mosaic virus	Rod shaped	Drug is conjugated to surface of virus nanoparticle	Doxorubicin, phenanthriplatin	Gulati *et al.* (2018)
Sesbania mosaic virus	Icosahedral	Fluorophore is conjugated to surface of virus particle	Fluorophores for imaging cancer	Vardhan *et al.* (2018)
Johnson grass chlorotic stripe mosaic virus	Icosahedral	Nanocarrier loaded with anticancer drug during *in vitro* assembly	Doxorubicin	Alemzadeh *et al.* (2017)
Potato virus X	Rod shaped	Drug is first bound to Potato virus X *in vitro* or delivered simultaneously along with naked Potato virus X nanoparticles	Doxorubicin	Lee *et al.* (2017)

## Cancer immunotherapy based on mathematical & computational modeling

A recent technical breakthrough in cancer immunotherapy has taken place with next-generation sequencing. This, in conjunction with computational methods, has hastened the processing of raw data to facilitate the mapping of mutations and the prediction of potential novel epitopes [39,40]. Plant virus nanoparticles can also undergo computational modeling to improve their immune response for cancer therapy [41–43]. Using computer simulations, researchers can make predictions as to the strength of plant virus coat protein interactions, and how these subunits can assemble under specific physiological conditions [44–46]. For example, Chariou *et al*. [47] used a mathematical model to explore the diffusion and cellular uptake of TMV in the form of a spheroid system rather than its native rod shape, in the presence and absence of modified surface chemistry. A combination of *in silico* and wet lab experiments can assist in the rational design of newly engineered VLPs that carry immunogenic peptides specific for tumors [48]. Alternatively, computational modeling can help design plant virus nanoparticles to be carriers for anticancer drugs [33,49].

## Nanoparticle design for cancer immunotherapy

The advent of computational biology is the beginning of a new era in vaccine technology. The design of a functional vaccine for cancer treatment through the use of computational biology is a demanding challenge. As breast cancer is one of the most common and deadliest of all cancers, it has become one of the most important candidates for vaccine development [50]. The use of computational methods not only reduces time and cost to design vaccines, it also increases efficiency of development. Epitope prediction, reverse, system and structural vaccinology are the byproducts of bioinformatics with respect to vaccine development [51].

The computational design of vaccines for breast cancer was made possible through the growing attention of researchers investigating immunological strategies to fight the development of breast cancer [52]. One technique includes elucidation of the sequence of the protein of interest, from which suitable epitopes for vaccine development can be selected from the database. Eventually, the selected epitopes are attached together to develop the final cancer vaccine. Plant virus-based vectors have recently been used for the production of vaccines against cancer, for example, spherical nanoparticles based on the rod-shaped TMV have been engineered by Bruckman *et al*. [53], loaded with Dox and used as a therapy against breast cancer. Similarly, Shukla *et al.* [54], expressed HER2 epitopes on the surface of CPMV and PVX nanoparticles that successfully raised Her2 antibody titres.

## Tumor-specific monoclonal antibodies

Monoclonal antibodies have been identified as a functional and efficient therapeutic agent for a number of malignancies [55]. These are among the most valuable and successful cancer therapeutics. While there is evidence that in certain instances monoclonal antibodies have the capability to damage the cancer cell wall, in others, they can interfere with cancer cell growth. Monoclonal antibodies can stimulate cells of the immune system or result in the self destruction of a cell [56].

Due to a monoclonal antibody’s potential to bind specifically to cancer cells, antibodies can be utilized to deliver treatments. For example, a monoclonal antibody can carry a small radioactive particle for radiation therapy to cancer cells with very little or no effect on nontarget cells, in a process called radioimmunotherapy. In the same manner, monoclonal antibodies can be attached to a chemotherapeutic medicine to selectively deliver it to cancer cells. Some can facilitate immune system targeting of cancer cells with the combination of two monoclonal antibodies; the first attaches to a cancer cell to flag it and the second to a specific immune cell to stimulate attack [57]. Plants have been efficiently used for the production of monoclonal antibodies, with tobacco being the first [58]. The use of plants is beneficial for their low production cost and ease of manipulation [59]. For example, Komarova *et al.* [60] generated the heavy and light chains of monoclonal antibodies against herceptin using TMV and PVX-based plant virus vectors in transgenic tobacco plants. Similarly, Esfandiari *et al.* [61] generated monoclonal antibodies (Mabs) specific for herceptin on the surface of PVX nanoparticles to target breast cancer. Monoclonal antibodies generated in plants that target cancer are summarized by Moussavou *et al.* [62].

## Next-generation immuno-oncology therapies

To advance and approve the next generation of immuno-oncology therapies, researchers must understand the process of identification of tumor cells by natural immune cells. Knowledge regarding how tumors are capable of avoiding immune cell attack will guide us to adopt novel, advanced strategies for cancer treatment. Plant virus-derived VLPs characterize a strategy for targeting the cell and delivering a therapeutic drug in a single step. Biologics such as drugs can be loaded onto the exterior or into the interior of plant viruses, thus rendering them highly effective oncolytic agents. While production cost is low in general, specialized requirements for standard purification will still be required. Steele [63] describe various cargos that plant virus nanoparticles can deliver to cancer cells for therapy or imaging.

## Future perspective

The field of cancer immunotherapy has never looked as promising as it does today. Currently, many exciting developments have taken place and our knowledge regarding how cancer evades the immune system has vastly improved. The safety and facile manipulation of plant virus-based VLPs could revolutionize the field of immunotherapy. Utilization of today’s mathematical modeling systems and computer technologies can improve the application of plant virus nanoparticles for cancer immunotherapy extensively. Increased understanding of cancer immunotherapy, combined with tools of nanotechnology and bioinformatics, has vastly improved our ability to fight cancer. These improvements will soon be reflected in the results of clinical trials.

**Executive summary**

- Nanotechnology is the growing science of today with promising impacts on nanomedicine.
- Plant virus-based nanoparticles have been shown to be effective for cancer treatment.
- Plant virus nanoparticles can be functionalized so that they can be preferentially taken up by cancer cells.
- Computational modeling can help design plant virus nanoparticles to be carriers for anticancer drugs.
- Plant virus-derived virus-like particles offer a low-cost strategy for targeting the cell and delivering a therapeutic drug.
